# Cerebral Blood Flow, Oxygen Delivery, and Pulsatility Responses to Oxygen Inhalation at High Altitude: Highlanders vs. Lowlanders

**DOI:** 10.3389/fphys.2019.00061

**Published:** 2019-02-07

**Authors:** Chang-Yang Xing, Jorge M. Serrador, Allan Knox, Li-Hua Ren, Ping Zhao, Hong Wang, Jie Liu

**Affiliations:** ^1^Department of Ultrasound Diagnostics, Tangdu Hospital, Fourth Military Medical University, Xi’an, China; ^2^Department of Pharmacology, Physiology and Neuroscience, New Jersey Medical School, Rutgers University, Newark, NJ, United States; ^3^Exercise Science Department, California Lutheran University, Thousand Oaks, CA, United States; ^4^General Hospital of Tibet Military Area Command, Lhasa, China; ^5^Department of Ultrasound Diagnostics, Jinling Hospital, Nanjing University School of Medicine, Nanjing, China

**Keywords:** hypobaric hypoxia, brain perfusion, middle cerebral artery, ultrasound, resistive index, pulsatility index, Tibetans, Han Chinese

## Abstract

**Objective:** To determine whether the acute cerebral hemodynamic responses to oxygen inhalation are impacted by race or acclimation to high altitude.

**Methods:** Three groups of young healthy males, who were Tibetans (highlanders, *n* = 15) with lifelong exposure to high altitude, and Han Chinese (lowlanders) with five-year (Han-5 yr, *n* = 15) and three-day (Han-3 d, *n* = 16) exposures, participated in the study at an altitude of 3658 m. Cerebral blood flow velocity (CBFV) was recorded for three minutes prior to and during pure oxygen inhalation (2 L/min), respectively, using a transcranial color-coded duplex (TCCD) sonography at the middle cerebral artery (MCA). The blood draw and simultaneous monitoring of blood pressure (BP), heart rate (HR), and finger arterial oxygen saturation (SaO_2_) were also performed.

**Results:** Values are Mean ± SEM. The three groups had similar demographic characteristics and HR responses, with the group differences (*P* < 0.05) found in hemoglobin concentration (16.9 ± 0.9, 18.4 ± 1.3, and 15.5 ± 1.0 gm/dL), baseline BPs and HR as expected. Both the Tibetans and Han-5yr groups presented blunted BP responses to O_2_-inhalation when compared to the Han-3d group; more interestingly, the Tibetans showed significantly reduced responses compared with Han-5yr and Han-3d in CBFV, cerebral oxygen delivery (COD), and pulsatility index (PI) as assessed by Δ%CBFV/ΔSaO_2_ (-1.50 ± 0.25 vs. -2.24 ± 0.25 and -2.23 ± 0.27, *P* = 0.049 and 0.048), Δ%COD/ΔSaO_2_ (-0.52 ± 0.27 vs. -1.33 ± 0.26 and -1.38 ± 0.28, *P* = 0.044 and 0.031), and Δ%PI (7 ± 2 vs. 16 ± 3 and 16 ± 3 %, *P* = 0.036 and 0.023), respectively.

**Conclusion:** These findings provide evidence on the Tibetans trait of a distinct cerebral hemodynamic regulatory pattern to keep more stable cerebral blood flow (CBF), oxygen delivery, and pulsatility in response to oxygen inhalation as compared with Han Chinese, which is likely due to a genetic adaptation to altitude.

## Introduction

The racial difference of hypoxic adaptive pattern between Tibetans and lowlanders at high altitude has been extensively studied. Previous studies indicate that Tibetans have lower resting blood pressure (BP), heart rate (HR), hemoglobin concentration (Hb) and cerebral blood flow (CBF), but higher resting ventilation and augmented acute hypoxic ventilatory responses in comparison with the immigrant lowlanders (Wu et al., 2005; Beall, 2007; Gilbert-Kawai et al., 2014; Liu et al., 2016). Although their findings provide insight into the physiological adaptation to a hypoxic environment, the contrary remains elusive as the cerebral hemodynamic responses to oxygen (O_2_) inhalation (O_2_-inhalation) in Tibetans compared with lowlanders is still unknown.

Our previous cross-sectional study indicates that Tibetans may have distinct cerebral hemodynamic responses to the long-term (> one year) altitude exposure (i.e., oxygen partial pressure change) when compared with Han Chinese (Liu et al., 2016). Additionally, recent studies suggest that oxygen therapy or conditioning may be used to counter the adverse effects of hypoxia on CBF and cognitive function in both the immigrant lowlanders and native residents at altitude (Yan, 2014; West, 2016a,b). Data addressing the potential differences in responses to O_2_-inhalation will provide foundations for optimizing the therapeutic potential of this intervention. Such data may also further our knowledge and understanding concerning the oxygen-related cerebral hemodynamic responses, years of acclimation in lowlanders, and genetic adaptation in Tibetans.

Therefore, the present study aimed to determine the acute cerebral hemodynamic response to O_2_-inhalation between Tibetans and immigrant lowlanders with short- or long-term (i.e., acute or chronic) high-altitude exposure. A secondary aim was to provide insight into the specific racial trait to high-altitude adaptation as suggested by our previous findings (Liu et al., 2016).

## Materials and Methods

### Subjects

A total of fifteen Tibetans and thirty-one Han Chinese signed informed consent to participate in the study. The Tibetan subjects were born and raised at the Qinghai-Tibetan Plateau (3240∼4507 m), while the Han subjects were born and raised close to sea level (≤ 400 m). Their ancestries were confirmed using family history questionnaires. None of the subjects had traveled to other altitudes within one year before participation. Participants were divided into three groups: (i) Tibetan natives with lifelong exposure to high altitude (Tibetans, *n* = 15); (ii) Han immigrants to high altitude for exactly five years (Han-5yr, *n* = 15), who were already acclimated to the altitude; and (iii) Han newcomers who were recently exposed to the high altitude for three days (Han-3d, *n* = 16). All data were collected at Lhasa (3658 m). This study was approved by the ethics committees of Tangdu hospital affiliated to Fourth Military Medical University and General Hospital of Tibet Military Area Command, which was performed in accordance with the guidelines of the *Declaration of Helsinki* and *Belmont Report*.

All subjects were young (17–27 years) and healthy as screened by medical history questionnaire and standard physical examination. None of the subjects had any known cerebrovascular, cardiovascular, or pulmonary disorders, nor did they have high-altitude illnesses, e.g., acute or chronic mountain sickness, high-altitude cerebral or pulmonary edema, as confirmed according to the international consensus statement (Leon-Velarde et al., 2005; Bartsch and Swenson, 2013). No subjects administered medication prior to or during the study. Subjects were required to refrain from calorific intake two hours before test and refrained from high-intensity exercise, alcohol, tea or caffeinated drinks for at least 24 h prior to data collection. The data collection was performed in a temperature-controlled room (23°C) with the subject in the left recumbent position following a blood draw and a 10-min rest. Physiological data were collected continuously for three minutes prior to and during continuous pure O_2_ inhalation (Note: post-O_2_ data collection started at the fourth minute of O_2_-inhalation). Subjects were asked to remain still, silent and awake throughout the test to optimize physiological stabilization and data collection.

### General Data Collection

During each stage, brachial systolic and diastolic BP (SBP and DBP) and HR were measured with an interval of ∼30 s using an electronic sphygmomanometer (HEM-7051, Omron Healthcare, Japan). Arterial blood oxygen saturation (SaO_2_) was measured simultaneously using a finger pulse oximeter (CMS50E, Contec Medical Systems, China). All of these parameters were measured three times with the averaged values used for final data analysis. Venous blood sample was drawn to assess hematocrit (Hct) and Hb.

### Transcranial Color-Coded Duplex Sonographic Measurement

A color-coded duplex ultrasonography system (Vivid 7, GE Vingmed Ultrasound AS, Horten, Norway) with a 1.8–3.4 MHz sector array probe (M3S-D) was used to record the CBFV (Bartels, 2012). With the subject in the left recumbent position, the right middle cerebral artery (MCA) was displayed through the temporal window by transcranial color-coded duplex (TCCD) sonography. The TCCD is more accurate than the transcranial Doppler aided by its visual angle correction function, but it still cannot measure the diameter of MCA due to inadequate spatial resolution. The Doppler sampling position and angle were adjusted according to the real-time image to achieve the optimal and most accurate signal.

The CBFV was recorded continuously for three minutes at the MCA prior to and during continuous pure O_2_ inhalation through a nasal cannula at 2 L/min. This is a routine dosage for low-flow oxygen therapy in clinics (Kearley et al., 1980) and could increase the fraction of inspired O_2_ from 21% (air) to approximately 28–30% in the present test (O’Reilly Nugent et al., 2014). All the TCCD measurements were performed by the same researcher (J. L.) who has over 10 years of experience in vascular ultrasound.

### Cerebral Hemodynamic Metrics

**FIGURE 1:**
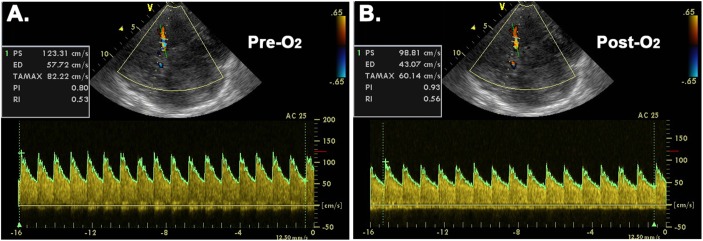
Representative transcranial color-coded duplex sonographic images before **(A)** and after **(B)** O_2_-inhalation. Pre-O_2_ = before O_2_-inhalation; Post-O_2_ = after O_2_-inhalation; AC = angle correction; PS = peak systolic velocity; ED = end diastolic velocity; TAMAX = time-averaged maximum velocity; PI = pulsatility index; RI = resistive index.

The peak systolic velocity (PSV), end diastolic velocity (EDV), time-averaged maximum velocity (defined as CBFV), resistive index (RI), and pulsatility index (PI) were obtained semi-automatically (Figure 1), where the RI = (PSV-EDV)/ PSV, and PI = (PSV-EDV)/CBFV. The time-averaged value of each parameter at each stage was used for final analysis. The mean arterial pressure (MAP) = (SBP+2 × DBP)/3, and the cerebrovascular resistance index (CVRi, mmHg ⋅ s/cm) = MAP/CBFV (Liu et al., 2013). The arterial content of oxygen (CaO_2_) was calculated as: CaO_2_ (ml O_2_/dL) = 1.36 (ml O_2_/g Hb) × Hb (g/dL) × SaO_2_ (%)/100, with the small quantity of dissolved oxygen (decreases further with rising altitude) not included in the estimation (Imray et al., 2014). To estimate the percentage change (Δ%) of cerebral oxygen delivery (COD) after O_2_-inhalation, the Δ%CBFV is assumed approximate to Δ%CBF in present study, as the COD (mL O_2_/min) can be calculated from the product of CBF and CaO_2_.

### Data Analysis

Statistical analysis was performed with the SPSS 19.0 software (IBM, Armonk, NY, United States). One-way ANOVA was used to compare the basic variables among the three groups, with the LSD test used to make *post hoc* pairwise group comparisons. The data distribution was assessed by the Shapiro–Wilk test. Homogeneity of variances was assessed by Levene’s test of homogeneity of variance. The repeated-measures ANOVA was performed to compare the physiological parameters pre and post O_2_-inhalation with the group differences examined in the responses (interaction effects). The potential impacts of baseline values on the hemodynamic responses to O_2_-inhalation were tested using correlation analysis between baseline and response variables, with no any significance observed. All data are presented as mean ± SEM, with *P* < 0.05 considered statistically significant.

## Results

### Demographic Characteristics and Baseline Hemodynamics

Table 1 summarizes demographic features and TCCD settings of all groups. All subjects increased their SaO_2_ to a stable level within four minutes. The body weight and body mass index of Tibetans and Han-5yr groups were comparable but both little lower than the Han-3d. The Tibetans had lower levels of Hct and Hb than the Han-5yr group, but slightly higher Hct and Hb than the Han-3d group. The Han-5yr group was approximately three to four years older than the other groups as expected, since most of the Han subjects were soldiers from sea-level with older ones staying longer at altitude. There were also no differences in the TCCD settings between groups.

**Table 1:** Demographic characteristics of the three male groups at high altitude.

Variables	Han- 3d	Han- 5yr	Tibetans	*P*
Sample size, *n*	16	15	15	
Age, years	18.8 ± 0.4^*^	23.0 ± 0.5^#^	19.8 ± 0.5	<0.001
Height, cm	173 ± 1	172 ± 1	170 ± 1	0.384
Weight, kg	67 ± 2^#^^*^	61 ± 1	60 ± 1	0.019
BMI, kg/m^2^	22.3 ± 0.6^#^^*^	20.7 ± 0.4	20.7 ± 0.4	0.021
Hct, %	47.4 ± 0.5^#^^*^	55.7 ± 0.9^#^	50.8 ± 0.7	<0.001
Hb, gm/dL	15.5 ± 0.2^#^^*^	18.4 ± 0.3^#^	16.9 ± 0.2	<0.001
Time to stable SaO_2_ after O_2_-inhalation, sec	72 ± 6	65 ± 9	80 ± 11	0.470
***TCCD settings***				
Angle correction, °	25 ± 5	20 ± 4	27 ± 4	0.419
SV depth, mm	52 ± 1	51 ± 2	51 ± 1	0.702


*Values are mean ± SEM. ^#^P < 0.05, vs. Tibetans; and ^∗^P < 0.05, Han-3d vs. Han-5yr. Han-3d = Han newcomers at high altitude for only3 days; Han-5yr = Han immigrants at high altitude for 5 years; Tibetans = Tibetan natives at high altitude; BMI = body mass index; Hct = hematocrit; Hb = hemoglobin concentration; SaO_2_ = arterial blood oxygen saturation; TCCD = transcranial color-coded duplex sonography; SV = sample volume.*

The baseline physiological values are presented in Table 2. The SaO_2_ was comparable between the Tibetans and Han-5yr groups, and lowest in the Han-3d group. BP (more significant for DBP) was generally lowest in the Tibetans and not different between Han groups. The CaO_2_ was significantly different between the three groups, with the Han-5yr being the highest and the Han-3d being the lowest. The baseline HR and CBFV showed no differences between the Tibetans and Han-5yr, but were highest in the Han-3d. Although the CVRi showed no significant differences between the three groups, the PI and RI were significantly higher in the Tibetans than in the Han-5yr and Han-3d groups, with no differences found between Han groups.

**Table 2:** Systemic and cerebral hemodynamics before and after continuous O_2_-inhalation in the three groups at high altitude.

Variables^§^	Han- 3d (*n* = 16)				Han- 5yr (*n* = 15)				Tibetans (*n* = 15)			
	Baseline	O_2_-inhalation	Δ	Δ%	Baseline	O_2_-inhalation	Δ	Δ%	Baseline	O_2_-inhalation	Δ	Δ%
***Systemic measurements***												
SaO_2_, %	85.7 ± 0.9^#^^*^	98.6 ± 0.1	13.0 ± 1.0^#^^*^	15 1^#^^*^	90.6 ± 0.3	98.7 ± 0.2	8.1 ± 0.3	9 ± 0	88.8 ± 0.7	98.4 ± 0.2	9.6 ± 0.7	11 ± 1
CaO_2_, ml O_2_/dL	18.1 ± 0.4^#^^*^	20.8 ± 0.3^#^^*^	2.7 ± 0.2^#^^*^	15 ± 1^#^^*^	22.6 ± 0.4^#^	24.6 ± 0.4^#^	2.0 ± 0.1	9 ± 0	20.4 ± 0.3	22.6 ± 0.3	2.2 ± 0.2	11 ± 1
SBP, mmHg	125 ± 3^#^^*^	113 ± 3^#^	-12 ± 2^#^^*^	-9 ± 1^#^^*^	112 ± 3	106 ± 2	-5 ± 1	-5 ± 1	110 ± 3	104 ± 2	-6 ± 2	-5 ± 1
DBP, mmHg	80 ± 3^#^	69 ± 3^#^	-12 ± 1^#^	-15 ± 2	75 ± 2^#^	65 ± 1^#^	-9 ± 1	-12 ± 1	65 ± 3	59 ± 2	-7 ± 1	-10 ± 2
MAP, mmHg	95 ± 3^#^^*^	83 ± 3^#^	-12 ± 1^#^^*^	-12 ± 1^#^	87 ± 2	79 ± 2	-8 ± 1	-9 ± 1	80 ± 2	74 ± 2	-6 ± 1	-8 ± 1
Heart rate, beats/min	79 ± 4^*^	68 ± 3^*^	-11 ± 2	-13 ± 2	65 ± 2	56 ± 1	-8 ± 2	-12 ± 2	70 ± 3	63 ± 3	-7 ± 1	-10 ± 2
***TCCD measurements***												
PSV, cm/s	130 ± 5^#^^*^	101 ± 5	-29 ± 2^#^^*^	-22 ± 2^#^^*^	109 ± 7	96 ± 6	-13 ± 2	-12 ± 2	113 ± 4	101 ± 3	-12 ± 2	-10 ± 2
EDV, cm/s	59 ± 3^#^^*^	41 ± 2	-18 ± 2^#^^*^	-30 ± 3^#^^*^	50 ± 3	40 ± 3	-10 ± 1	-20 ± 2	46 ± 2	39 ± 1	-7 ± 1	-14 ± 2
CBFV, cm/s	82 ± 3^#^^*^	60 ± 3	-22 ± 2^#^^*^	-27 ± 2^#^^*^	70 ± 4	58 ± 4	-12 ± 1	-17 ± 1	67 ± 2	58 ± 2	-9 ± 1	-13 ± 2
CVRi, mmHg ⋅ s/cm	1.19 ± 0.06	1.43 ± 0.07	0.24 ± 0.03^#^^*^	21 ± 3^#^^*^	1.30 ± 0.07	1.44 ± 0.09	0.14 ± 0.03	11 ± 3	1.22 ± 0.05	1.29 ± 0.03	0.07 ± 0.03	7 ± 3
RI	0.55 ± 0.02^#^	0.60 ± 0.01	0.05 ± 0.01^#^	10 ± 2^#^	0.54 ± 0.01^#^	0.58 ± 0.01	0.04 ± 0.01	9 ± 2	0.60 ± 0.01	0.61 ± 0.01	0.02 ± 0.01	3 ± 1
PI	0.89 ± 0.04^#^	1.03 ± 0.04	0.14 ± 0.03^#^	16 ± 3^#^	0.85 ± 0.04^#^	0.97 ± 0.04	0.12 ± 0.02	16 ± 3^#^	1.02 ± 0.04	1.08 ± 0.04	0.06 ± 0.02	7 ± 2
COD, mL O_2_/min				-16 ± 2^#^^*^				-10 ± 2^#^				-4 ± 2


*^§^ Values are mean ± SEM. ^#^P < 0.05, vs. Tibetans; and ^∗^P < 0.05, Han-3d vs. Han-5yr. Han-3d = Han newcomers at high altitude for only 3 days; Han-5yr = Han immigrants at high altitude for 5 years; Tibetans = Tibetan natives at high altitude; Δ = absolute change from the baseline; Δ% = relative percentage change from the baseline; SaO_2_ = arterial blood oxygen saturation; CaO_2_ = arterial oxygen content; SBP = systolic blood pressure; DBP = diastolic blood pressure; MAP = mean arterial pressure; TCCD = transcranial color-coded duplex sonography; ICA = internal carotid artery; PSV = peak systolic velocity; EDV = end-diastolic velocity; CBFV = cerebral blood flow velocity; CVRi = cerebrovascular resistance index; RI = resistive index; PI = pulsatility index; COD = cerebral oxygen delivery.*

### Impact of O_2_-Inhalation on Systemic Hemodynamics

**FIGURE 2:**
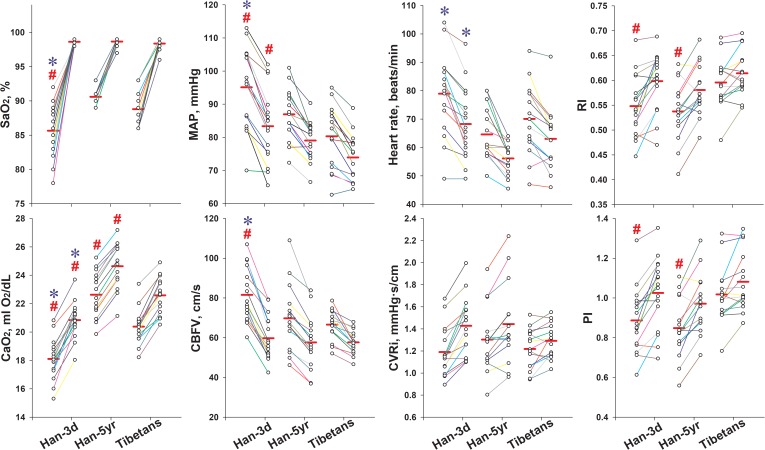
Individual values of key variables pre- and post O_2_-inhalation in the three groups. The left column of dots of each group were data before O_2_-inhalation, and the right ones were data after O_2_-inhalation. The solid red line in each column represents the mean value. All the presented variables changed significantly following O_2_-inhalation in all groups. ^#^*P* < 0.05, vs. Tibetans; and ^∗^*P* < 0.05, Han-3d vs. Han-5yr. Han-3d = Han newcomers at high altitude for only 3 days; Han-5yr = Han immigrants at high altitude for 5 years; Tibetans = Tibetan natives at high altitude; SaO_2_ = arterial blood oxygen saturation; CaO_2_ = arterial oxygen content; MAP = mean arterial pressure; CBFV = cerebral blood flow velocity; CVRi = cerebrovascular resistance index; RI = resistive index; PI = pulsatility index.

All measured physiological parameters after O_2_-inhalation are presented in Figure 2. The SaO_2_, CaO_2_, CVRi, RI, and PI increased, along with the decrease of BPs, HR, and CBFV in all groups (Table 2).

The induced changes of SaO_2_ and CaO_2_ were smaller in the Tibetans and Han-5yr groups than in the Han-3d group (ΔSaO_2_, 9.6 ± 0.7 and 8.1 ± 0.3 vs. 13.0 ± 1.0%; ΔCaO_2_, 2.2 ± 0.2, and 2.0 ± 0.1 vs. 2.7 ± 0.2 ml O_2_/dL, *P* < 0.05, respectively). The Tibetans and Han-5yr groups demonstrated reduced magnitudes of changes in SBP and MAP than Han-3d group in response to O_2_-inhalation (ΔSBP, -6 ± 2 and -5 ± 1 vs. -12 ± 2; ΔMAP, -6 ± 1, and -8 ± 1 vs. -12 ± 1 mmHg, *P* < 0.05, respectively). No significant differences were found for all measured systemic responses to O_2_-inhalation between the Tibetans and Han-5yr groups (Table 2). The HR response to O_2_-inhalation showed no significant differences between all groups.

### Impact of O_2_-Inhalation on Cerebral Hemodynamics

The Tibetans and Han-5yr groups showed decreased responses to O_2_-inhalation in PSV, EDV, CBFV, and CVRi than the Han-3d group. The Tibetans also demonstrated significantly reduced responses in PI and RI than Han-3d group (Δ%PI, 7 ± 2 vs. 16 ± 3, *P* = 0.023; Δ%RI, 3 ± 1 vs. 10 ± 2, *P* = 0.021). The Tibetans showed trends of smaller responses in CBFV (Δ%CBFV, -13 ± 2 vs. -17 ± 1, *P* = 0.071) and RI (Δ%RI, 3 ± 1 vs. 9 ± 2, *P* = 0.059), but significantly smaller responses in COD (Δ%COD, -4 ± 2 vs. -10 ± 2, *P* = 0.022) and PI (Δ%PI, 7 ± 2 vs. 16 ± 3, *P* = 0.036) compared to Han-5yr group; Moreover, the COD response in Han-5yr was smaller than in Han-3d (Δ%COD, -10 ± 2 vs. -16 ± 2, *P* < 0.001), with the PI and RI responses showed no significant differences between the two Han groups (Table 2).

**FIGURE 3:**
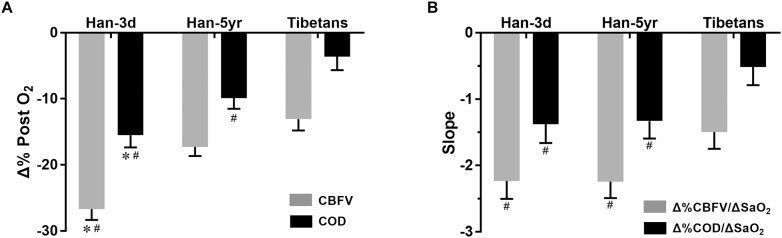
Column graphs of percentage changes of CBFV and COD post O_2_-inhalation **(A)** and their normalized values (i.e., their slopes when divided by the changes of SaO_2_) **(B)** in the three groups. The column height represents mean value, and the bar shows the SEM. ^#^*P* < 0.05, vs. Tibetans; and ^∗^*P* < 0.05, Han-3d vs. Han-5yr. Han-3d = Han newcomers at high altitude for only3 days; Han-5yr = Han immigrants at high altitude for 5 years; Tibetans = Tibetan natives at high altitude; CBFV = cerebral blood flow velocity; COD = cerebral oxygen delivery; SaO_2_ = arterial blood oxygen saturation; Δ%CBFV/ΔSaO_2_ = percentage change of CBFV (relative to baseline) per absolute change of SaO_2_, and so on.

More interestingly, the Tibetans showed significantly lower Δ%CBFV and Δ%COD per unit change of SaO_2_, i.e., Δ%CBFV/ΔSaO_2_ (-1.50 ± 0.25 vs. -2.24 ± 0.25 and -2.23 ± 0.27, *P* = 0.049 and 0.048) and Δ%COD/ΔSaO_2_ (-0.52 ± 0.27 vs. -1.33 ± 0.26 and -1.38 ± 0.28, *P* = 0.044 and 0.031), than both the Han-5yr and Han-3d groups (Figure 3), with no significant differences found in them between the two Han groups.

## Discussion

To the best of our knowledge, this is the first study to explore the impact of race (i.e., genetic adaptation) on cerebral hemodynamic responses to O_2_-inhalation at altitude. This investigation provides four novel findings; (i) the Tibetans and the acclimated Han immigrants showed blunted systemic hemodynamic responses to O_2_-inhalation compared with Han newcomers at altitude; (ii) the Tibetans also presented a blunted the COD response to O_2_-inhalation as compared to either acclimated Han immigrants or Han newcomers at altitude; (iii) when normalized by ΔSaO_2_, both CBF and COD responses were blunted in the Tibetans compared with both Han groups; (iv) the increase of CBF pulsatility to O_2_-inhalation was dampened in the Tibetans as compared to both Han groups.

### The Effects of Race and Acclimation on Resting Hemodynamics at Altitude

The resting hemodynamic characteristics of the Tibetans were basically consistent with previous reports, in slightly lower BP (Liu et al., 2016; Dhar et al., 2018), lower Hb (Beall et al., 1998; Li et al., 2017), but comparable CBFV (Smirl et al., 2014), as compared to the acclimated Han immigrants at altitude. These data collectively indicate Tibetans possess a unique physiological adaptive pattern to hypoxia at altitude, which features a more stable hemodynamic status without compensatory increase of hemoglobin level. The resting PI showed no difference between Han Chinese groups with acute and chronic exposures to altitude, but it was significantly higher in the Tibetans, implying that the increased resting CBF pulsatility might be a racial trait of cerebral hemodynamics in Tibetans.

The resting hemodynamic traits of Han Chinese groups with acute and chronic exposures to altitude agree with previous reports (Calbet, 2003; Liu et al., 2016), which suggests that BP and HR can be decreased in Han immigrants after years of acclimation as compared to their levels during acute exposure to altitude. This is likely associated with the autonomic cardiovascular acclimation to altitude, as reflected by an adjustment in sympathetic dominance and parasympathetic response (Malhotra et al., 1976; Dhar et al., 2018). The CBFV difference between Han-3d and Han-5yr in this study was also consistent with previous findings that the CBF of lowlanders at altitude tended to decrease back to its sea-level value after years of acclimation (Moller et al., 2002; Liu et al., 2016). This might be partly explained by a subsequent onset of ventilatory acclimation in lowlanders after acute exposure to altitude, i.e., the increase in partial pressure of arterial oxygen and decrease in partial pressure of arterial carbon dioxide due to hyperventilation (Rahn and Otis, 1949), leading to a reduction in CBF (Willie et al., 2012). Additionally, similar to previous investigations (Liu et al., 2016; Li et al., 2017), the Hb and Hct increased along with a time-course hemoglobin-oxygen affinity change in the Han population which reflects a physiological acclimation to maintain the CaO_2_ under the hypobaric hypoxic environment at altitude.

### The Effects of Race and Acclimation on Systemic Hemodynamic Responses to O_2_-Inhalation at Altitude

The decreased BP, HR, and CBFV, along with the increased CVRi in response to O_2_-inhalation at altitude were basically opposing responses as observed during acute exposure to hypoxia (Ainslie and Ogoh, 2010; Willie et al., 2012; Liu et al., 2016). The magnitudes of the systemic hemodynamic responses were comparable between the Tibetans and Han-5yr, but significantly lower (excluding the HR response) than in Han-3d at altitude. This finding, in combination with the similar baseline resting systemic hemodynamics between the Tibetans and Han-5yr as mentioned above, and similar baseline ventilation status (i.e., end-tidal CO_2_ and O_2_) between the Sherpas (a branch of Tibetans) and acclimated lowlanders as reported previously (Smirl et al., 2014), collectively indicate that the lowlanders can develop comparable systemic O_2_-inhalation responses to those of Tibetans after years of acclimation. The Han-5yr showed blunted systolic BP response but similar HR response to O_2_-inhalation compared to Han-3d indicate that the cardiovascular autonomic function is likely modified in the lowlanders after acclimation to altitude.

### The Effects of Race and Acclimation on Cerebral Hemodynamic Responses to O_2_-Inhalation at Altitude

The most interesting finding in present study is that the distinctive racial traits found in the cerebral but not in the systemic hemodynamic responses to O_2_-inhalation, i.e., the Tibetans and acclimated Han immigrants showed no significant differences of systemic hemodynamic changes as discussed above, but with the magnitudes of CBFV (normalized by ΔSaO_2_), COD, and PI responses found different between the two races. The Tibetans showed generally blunted CBF, oxygen delivery, and pulsatility responses to O_2_-inhalation than the acclimated Han immigrants at altitude, which would be more obvious when normalized by ΔSaO_2_. The blunted COD response is in line with our previous finding on a blunted total COD change of Tibetans as compared to Han Chinese for long-term altitude exposure from sea-level (0% vs. 10%) (Liu et al., 2016). The collective findings from the two studies suggest that the Tibetans possess a distinct cerebral hemodynamic regulatory pattern to keep more stable COD in response to surrounding oxygen variations than lowlanders, which is likely due to the genetic adaptation as a result of natural selection for thousands of years (Zhang et al., 2018). This race-specific ability may facilitate a better reserve on cerebral oxygen transport (Gilbert-Kawai et al., 2014). In addition, this blunted O_2_-inhalation response indicated the same dosage of oxygen therapy or conditioning may not function identically on the Tibetans and lowlanders at altitude, i.e., different individuals and races are likely to have their own optimal dosages of O_2_-inhalation to maximize the oxygen delivery to the brain. Thus our findings also suggest that racial differences in response to O_2_-inhalation should be taken into account when using oxygen therapy to treat high-altitude diseases (Leon-Velarde et al., 2005; Bartsch and Swenson, 2013), or oxygen conditioning to improve the living and working conditions of people at altitude (West, 2017). Similarly, the possible blunted cerebral hemodynamic responses to O_2_-inhalation should also be considered when considering oxygen therapy for highlanders at low altitude.

Additionally, the decreased responses of CBFV, CVRi, and COD, but similar RI and PI responses to O_2_-inhalation in the Han-5yr compared with Han-3d indicates lowlanders likely blunt their CBF and oxygen delivery responses but with pulsatility response unaffected after acclimation.

### Strengths and Limitations

The major strength of this study is the integrated design of racial and acclimation effects on hemodynamic responses to O_2_-inhalation. This design enabled a comparison between genetic adaptation and years of acclimation at altitude. In addition, all the subjects were young and healthy with very similar backgrounds and demographic features except for the high-altitude experience; and their group differences were representative in hemoglobin and baseline physiological values at altitude, which are consistent with previous reports (Pawson, 1977; Gilbert-Kawai et al., 2014; Liu et al., 2016).

There are several limitations that should be mentioned. Firstly, we used the CBFV at MCA to estimate the CBF, which did not consider the potential vasoconstrictive effect of O_2_-inhalation on MCA diameter. Although the TCCD measurements at MCA have been suggested as a valid method to estimate CBF changes during acute changes of arterial blood gasses (Willie et al., 2012), it still may lead to an underestimation of the CBF reductions during O_2_-inhalation in our study. Secondly, the absence of end-tidal CO_2_ (ETCO_2_) or PaCO_2_ limited our interpretation of CBFV response (Markwalder et al., 1984). However, the O_2_-inhalation was reported to decrease ETCO_2_ in the Tibetan group but not in the acclimated Han immigrants (Zhuang et al., 1993), suggesting that Tibetans would respond with a hypocapnic cerebral vasoconstriction (Norcliffe et al., 2005) while the acclimated lowlanders would not. In this regard, the smaller CBFV decrease in the Tibetans in contrast with Han-5yr following O_2_-inhalation might be even more significant if the ETCO_2_ level could be clamped. Thirdly, the CBFV was measured only at MCA, which makes our results specific to the anterior cerebral circulation and unable to be extrapolated to the whole brain. It is still unknown whether there are regional differences (anterior vs. posterior circulation) in the cerebral hemodynamic responses to O_2_-inhalation at altitude (Ogoh et al., 2013; Lewis et al., 2014; Feddersen et al., 2015). Finally, the present study only enrolled male subjects with the sex differences meriting further investigation.

## Conclusion

The Tibetans demonstrated a racial trait of blunted cerebral hemodynamic responses to O_2_-inhalation when compared with both Han newcomers and acclimated Han immigrants at altitude. Our results suggest the Tibetans possess a distinct cerebral hemodynamic regulatory pattern to keep more stable CBF, oxygen delivery, and pulsatility in response to acute variations of oxygen environment than lowlanders, as a result of altitude adaptation for thousands of years.

## Author Contributions

C-YX conducted the data analysis, interpretation, and wrote the manuscript. L-HR helped with subjects’ enrollment and data collection. JS and AK helped with data analysis, interpretation, and manuscript preparation. PZ and HW helped with data collection and manuscript preparation. JL designed the study, and performed the data collection, analysis, interpretation, and manuscript preparation. All authors edited and revised the manuscript and approved final submission.

## Conflict of Interest Statement

The authors declare that the research was conducted in the absence of any commercial or financial relationships that could be construed as a potential conflict of interest.

## References

[B1] Ainslie P. N. Ogoh S. (2010). Regulation of cerebral blood flow in mammals during chronic hypoxia: a matter of balance. *Exp. Physiol.* 95 251–262. 10.1113/expphysiol.2008.045575 19617269

[B2] Bartels E. (2012). Transcranial color-coded duplex ultrasonography in routine cerebrovascular diagnostics. *Perspect. Med.* 1 325–330. 10.1016/j.permed.2012.06.001 12470850

[B3] Bartsch P. Swenson E. R. (2013). Acute high-altitude illnesses. *N. Engl. J. Med.* 369 1666–1667. 10.1056/NEJMc1309747 24152275

[B4] Beall C. M. (2007). Two routes to functional adaptation: tibetan and andean high-altitude natives. *Proc. Natl. Acad. Sci. U.S.A.* 104(Suppl. 1), 8655–8660. 10.1073/pnas.0701985104 17494744 PMC1876443

[B5] Beall C. M. Brittenham G. M. Strohl K. P. Blangero J. Williams-Blangero S. Goldstein M. C. (1998). Hemoglobin concentration of high-altitude tibetans and bolivian Aymara. *Am. J. Phys. Anthropol.* 106 385–400. 10.1002/(SICI)1096-8644(199807)106:3<385::AID-AJPA10>3.0.CO;2-X 9696153

[B6] Calbet J. A. (2003). Chronic hypoxia increases blood pressure and noradrenaline spillover in healthy humans. *J. Physiol.* 551(Pt 1), 379–386. 10.1113/jphysiol.2003.045112 12844510 PMC2343162

[B7] Dhar P. Sharma V. K. Das S. K. Barhwal K. Hota S. K. Singh S. B. (2018). Differential responses of autonomic function in sea level residents, acclimatized lowlanders at > 3500m and Himalayan high altitude natives at >3500m: a cross-sectional study. *Respir. Physiol. Neurobiol.* 254 40–48. 10.1016/j.resp.2018.04.002 29649580

[B8] Feddersen B. Neupane P. Thanbichler F. Hadolt I. Sattelmeyer V. Pfefferkorn T. (2015). Regional differences in the cerebral blood flow velocity response to hypobaric hypoxia at high altitudes. *J. Cereb. Blood Flow Metab.* 35 1846–1851. 10.1038/jcbfm.2015.142 26082017 PMC4635241

[B9] Gilbert-Kawai E. T. Milledge J. S. Grocott M. P. Martin D. S. (2014). King of the mountains: tibetan and sherpa physiological adaptations for life at high altitude. *Physiology* 29 388–402. 10.1152/physiol.00018.2014 25362633

[B10] Imray C. Chan C. Stubbings A. Rhodes H. Patey S. Wilson M. H. (2014). Time course variations in the mechanisms by which cerebral oxygen delivery is maintained on exposure to hypoxia/altitude. *High Alt. Med. Biol.* 15 21–27. 10.1089/ham.2013.1079 24559404

[B11] Kearley R. Wynne J. W. Block A. J. Boysen P. G. Lindsey S. Martin C. (1980). The effect of low flow oxygen on sleep-disordered breathing and oxygen desaturation. A study of patients with chronic obstructive lung disease. *Chest* 78 682–685. 10.1378/chest.78.5.682 7428451

[B12] Leon-Velarde F. Maggiorini M. Reeves J. T. Aldashev A. Asmus I. Bernardi L. (2005). Consensus statement on chronic and subacute high altitude diseases. *High Alt. Med. Biol.* 6 147–157. 10.1089/ham.2005.6.147 16060849

[B13] Lewis N. C. Messinger L. Monteleone B. Ainslie P. N. (2014). Effect of acute hypoxia on regional cerebral blood flow: effect of sympathetic nerve activity. *J. Appl. Physiol.* 116 1189–1196. 10.1152/japplphysiol.00114.2014 24610534 PMC4098059

[B14] Li C. Li X. Liu J. Fan X. You G. Zhao L. (2017). Investigation of the differences between the tibetan and han populations in the hemoglobin-oxygen affinity of red blood cells and in the adaptation to high-altitude environments. *Hematology* 23 309–313. 10.1080/10245332.2017.1396046 29130390

[B15] Liu J. Liu Y. Ren L. H. Li L. Wang Z. Liu S. S. (2016). Effects of race and sex on cerebral hemodynamics, oxygen delivery and blood flow distribution in response to high altitude. *Sci. Rep.* 6:30500. 10.1038/srep30500 27503416 PMC4977556

[B16] Liu J. Zhu Y.-S. Hill C. Armstrong K. Tarumi T. Hodics T. (2013). Cerebral autoregulation of blood velocity and volumetric flow during steady-state changes in arterial pressure. *Hypertension* 62 973–979. 10.1161/HYPERTENSIONAHA.113.01867 24041946 PMC3893684

[B17] Malhotra M. S. Selvamurthy W. Purkayastha S. S. Mukherjee A. K. Mathew L. Dua G. L. (1976). Responses of the autonomic nervous system during acclimatization tp high altitude in man. *Aviat. Space Environ. Med.* 47 1076–1079. 985281

[B18] Markwalder T. M. Grolimund P. Seiler R. W. Roth F. Aaslid R. (1984). Dependency of blood flow velocity in the middle cerebral artery on end-tidal carbon dioxide partial pressure–a transcranial ultrasound Doppler study. *J. Cereb. Blood Flow Metab.* 4 368–372. 10.1038/jcbfm.1984.54 6432808

[B19] Moller K. Paulson O. B. Hornbein T. F. Colier W. N. Paulson A. S. Roach R. C. (2002). Unchanged cerebral blood flow and oxidative metabolism after acclimatization to high altitude. *J. Cereb. Blood Flow Metab.* 22 118–126. 10.1097/00004647-200201000-00014 11807401

[B20] Norcliffe L. J. Rivera-Ch M. Claydon V. E. Moore J. P. Leon-Velarde F. Appenzeller O. (2005). Cerebrovascular responses to hypoxia and hypocapnia in high-altitude dwellers. *J. Physiol.* 566(Pt 1), 287–294. 10.1113/jphysiol.2005.086629 15860531 PMC1464723

[B21] Ogoh S. Sato K. Nakahara H. Okazaki K. Subudhi A. W. Miyamoto T. (2013). Effect of acute hypoxia on blood flow in vertebral and internal carotid arteries. *Exp. Physiol.* 98 692–698. 10.1113/expphysiol.2012.068015 23143991

[B22] O’Reilly Nugent A. Kelly P. T. Stanton J. Swanney M. P. Graham B. Beckert L. (2014). Measurement of oxygen concentration delivered via nasal cannulae by tracheal sampling. *Respirology* 19 538–543. 10.1111/resp.12268 24661379

[B23] Pawson I. G. (1977). Growth characteristics of populations of tibetan origin in Nepal. *Am. J. Phys. Anthropol.* 47 473–482. 10.1002/ajpa.1330470320 201173

[B24] Rahn H. Otis A. B. (1949). Man’s respiratory response during and after acclimatization to high altitude. *Am. J. Physiol.* 157 445–462. 10.1152/ajplegacy.1949.157.3.445 18151752

[B25] Smirl J. D. Lucas S. J. Lewis N. C. Dumanoir G. R. Smith K. J. Bakker A. (2014). Cerebral pressure-flow relationship in lowlanders and natives at high altitude. *J. Cereb. Blood Flow Metab.* 34 248–257. 10.1038/jcbfm.2013.178 24169852 PMC3915197

[B26] West J. B. (2016a). Cognitive impairment of school children at high altitude: the case for oxygen conditioning in schools. *High Alt. Med. Biol.* 17 203–207. 10.1089/ham.2016.0026 27355278

[B27] West J. B. (2016b). Oxygen conditioning: a new technique for improving living and working at high altitude. *Physiology* 31 216–222. 10.1152/physiol.00057.2015 27053735

[B28] West J. B. (2017). Are permanent residents of high altitude fully adapted to their hypoxic environment? *High Alt. Med. Biol.* 18 135–139. 10.1089/ham.2016.0152 28256920

[B29] Willie C. K. Macleod D. B. Shaw A. D. Smith K. J. Tzeng Y. C. Eves N. D. (2012). Regional brain blood flow in man during acute changes in arterial blood gases. *J. Physiol.* 590 3261–3275. 10.1113/jphysiol.2012.228551 22495584 PMC3459041

[B30] Wu T. Wang X. Wei C. Cheng H. Wang X. Li Y. (2005). Hemoglobin levels in Qinghai-Tibet: different effects of gender for tibetans vs. han. *J. Appl. Physiol.* 98 598–604. 10.1152/japplphysiol.01034.2002 15258131

[B31] Yan X. (2014). Cognitive impairments at high altitudes and adaptation. *High Alt. Med. Biol.* 15 141–145. 10.1089/ham.2014.1009 24949527

[B32] Zhang X. L. Ha B. B. Wang S. J. Chen Z. J. Ge J. Y. Long H. (2018). The earliest human occupation of the high-altitude Tibetan Plateau 40 thousand to 30 thousand years ago. *Science* 362 1049–1051. 10.1126/science.aat8824 30498126

[B33] Zhuang J. Droma T. Sun S. Janes C. Mccullough R. E. Mccullough R. G. (1993). Hypoxic ventilatory responsiveness in tibetan compared with Han residents of 3,658 m. *J. Appl. Physiol.* 74 303–311. 10.1152/jappl.1993.74.1.303 8444707

